# Lessons learned for animal health governance from bovine viral diarrhea eradication schemes in Scotland and Ireland

**DOI:** 10.3389/fvets.2022.956635

**Published:** 2022-10-10

**Authors:** Orla Shortall

**Affiliations:** James Hutton Institute, Aberdeen, United Kingdom

**Keywords:** bovine viral diarrhea (BVD), Ireland, Scotland, interviews (qualitative), governance, cattle, beef, dairy

## Abstract

This paper explores lessons learned for animal health governance from bovine viral diarrhea (BVD) eradication schemes in Scotland and Ireland, drawing on qualitative key stakeholder interviews. Bovine viral diarrhea is an endemic cattle disease that causes animal health and welfare problems, as well as financial losses to farmers. Initial voluntary industry-led schemes to eradicate BVD were introduced in both countries in the 2010s, followed by compulsory phases involving legislation. The paper uses a theoretical framework of co-productive governance to analyze stakeholder views on how well the design and execution of the eradication schemes worked and what can be learned to inform future directions of animal health governance. The term “co-productive governance” comes from the field of environmental governance and was developed to describe how science and politics influence each other in a context where governance is carried out by multiple actors working collaboratively. The results of key stakeholder interviews are analyzed using the concepts of vision, context, knowledge, and process. In relation to vision, the results show the importance of creating a clear narrative about the goal of disease eradication schemes, which may incorporate or replace existing vet or farmer “narratives” about a disease. With regard to context, it is difficult to engage all actors in biosecurity governance, when initiatives are developed with the legacy of existing relationships and tensions. In relation to knowledge, the results showed the importance but political complexity of basing decisions on scientific research. One of the lessons learned was the benefit of involving industry stakeholders in setting scientific questions to inform the design of the scheme. Additionally, with reference to the process, while interviewees were enthusiastic about future prospects for industry and government working together to achieve biosecurity goals co-productive governance is not a panacea for enrolling all actors in biosecurity goals. The results also highlighted that farmers and other actors might object to an eradication scheme, whether it is run by government or private industry. Thus, it is useful to keep questions about who benefits in what way from biosecurity governance open.

## Introduction

This paper explores lessons learned for animal health governance from bovine viral diarrhea (BVD) eradication schemes in Scotland and Ireland, drawing on qualitative key stakeholder interviews. The paper uses a theoretical framework of co-productive governance to analyze stakeholder views on how well the design and execution of the eradication schemes worked and what can be learned to inform future directions of animal health governance. The term “co-productive governance” comes from the field of environmental governance and was developed to describe how science and politics influence each other in a context where governance is carried out by multiple actors working collaboratively (1). The paper uses Wyborn's (1) framework for analyzing co-productive governance in terms of context, vision, knowledge, and process.

Bovine viral diarrhea is an endemic cattle disease that causes animal health and welfare problems, as well as financial losses to farmers (2). BVD is primarily spread by persistently infected (PI) animals who are infected with the virus *in utero* (3). Cattle can also develop transient BVD infections. PI animals do not develop immunity to the disease and so shed viruses throughout their lifetime, infecting other animals they come into contact with. Research has shown that PI animals have worse health and productivity outcomes than non-PI animals (4). Eliminating PI animals was identified as the cornerstone of successful BVD eradication by a thematic network funded by the European Commission (5). Several successful eradication schemes have been carried out in European countries in recent decades, initially in Norway, Denmark, Sweden, and Finland in the 1990s and Germany, Austria, and Switzerland in the 2000s (6, 7). These countries achieved freedom from disease after around 10 years of eradication. The EU Animal Health Law regulation 2016/429 amended in 2021 brought BVD into EU legislation for the first time and allows countries to gain recognition for the BVD eradication scheme and sets criteria for the definition of BVD freedom. For a country to be recognized as free from BVD at least 99.8% of herds comprising at least 99.9% of the cattle population must be free from BVD. Countries that are recognized as free from BVD can implement trade barriers for cattle imported from countries that are not BVD free.

In this study, the BVD eradication schemes in the Republic of Ireland (hereafter referred to as Ireland) and Scotland were chosen for analysis because their different approaches to eradication and governance mechanisms make for a useful comparison. A voluntary BVD eradication scheme was introduced in Scotland in 2010, which provided funding for farmers to carry out BVD tests in their herds. Initially, farmers could choose from several tests: an antibody bulk milk test, an antigen ear tag test administered to all calves born on the farm, or a blood sample antibody test of five calves in each management group. In 2013 testing was made compulsory for breeding herds and in 2014 a requirement was put in place for breeding herds to have herd level or individual animal BVD negative status in order for animals to be sold. The fourth phase involved further testing requirements and movement restrictions and the fifth phase introduced in 2019 brought in additional measures for not negative or positive herds. A voluntary scheme was introduced in Ireland in 2012, using only one testing method: tissue tagging calves born on the farm. Testing was made compulsory in 2013 and movement restrictions were introduced in 2017 for herds with a positive or unknown status. Herds with a positive status also undergo a herd investigation, carried out by a vet, to identify the PI. There is a financial incentive paid to farmers for the prompt removal of PI animals within a given time frame.

This paper will use a framework of co-productive governance to analyze key stakeholder views of lessons learned from the BVD eradication schemes in Scotland and Ireland using the concepts of context, vision, process, and knowledge. There has been a trend in several countries, including Scotland and Ireland, of more shared responsibility for biosecurity between public and private bodies in recent decades (8). The concept of shared responsibility for biosecurity is under theorized in terms of how it is organized and what factors contribute to successfully sharing responsibility in biosecurity governance (8). The concept of co-productive governance (1) is used in this paper as a way to bring conceptual insights from other disciplines to biosecurity governance.

## Theoretical framework

### Co-production

Governance is defined as “the act or process of governing or overseeing the control and direction of something (such as a country or organization)” (9). The concept of co-production has roots in different disciplines. In relation to governance, the term was originally used in the late 1970s by the economist Elinor Ostrom to describe the involvement of citizens in the provision of public services (10). Citizens can use their skills, time, and other resources to coproduce the services they avail of, which can lead to better public service provision outcomes in certain circumstances (10). The idea of co-production was developed further by activists and academics to describe the involvement of a range of actors in governance (11). Co-productive decision making or “co-management” can be challenging as it can involve creating novel institutions with their own social norms and rules, and navigating competing interests and values in decision making (12).

Another related meaning of the concept of co-production is from the science and technology studies scholar Sheila Jasanoff who uses the term as a framework to investigate how science and society influence each other. “Increasingly, the realities of human experience emerge as the joint achievements of scientific, technical and social enterprises: science and society, in a word, are *coproduced* each underwriting the other's existence. [(13) p. 17 italics in original]. Science and technology studies investigate how science is a social endeavor carried out by people situated within particular institutional settings. Scientists operate within the social world and bring their personal and wider societal values to bear in their study (14). The fact that science is not value free is recognized in the context of animal health research (13). The intersection of science and policy is complicated because there is always a degree of indeterminacy and uncertainty in scientific findings which means they cannot map directly onto one course of political action (15). The government might use science “instrumentally” to answer particular research questions that inform policy (16). Research has also shown in practice that science is also used in a symbolic way to legitimize decisions, establish an actor's authority and substantiate policy positions (16). Additionally, scientists themselves can take differing roles, advocating for one policy, or presenting multiple options to policy makers (17). Because of the important but complicated relationship between governance and science, debates can become “scientized” when discussion about values and politics get displaced to the realm of science, with one side arguing for one body of scientific research and another highlighting the uncertainty in the science or championing an opposing body of research (15).

There is another, again related, the meaning of co-production which involves bringing together different types and sources of knowledge in the understanding of an issue: “This perspective leads into the realm of knowledge co-production, which we define as the collaborative process of bringing a plurality of knowledge sources and types together to address a defined problem and build an integrated or systems-oriented understanding of that problem.” [(18) p. 996]. This is a normative concept that maintains that the involvement of multiple actors in knowledge production enhances decision making (19).

The concept of co-production is intended to help us think through and analyze the connections between science and society, scientists and policy. Wyborn (1) develops a framework of “co-productive governance' drawing on adaptive governance and co-production literature which I will use in this paper to structure the analysis. Wyborn's (1) concept of co-productive governance expresses a normative aspiration of how knowledge and governance should interact: “Thus, co-production requires a fundamental transformation of both science and governance toward more critical, inclusive, and reflexive practice.” (1 p. 59)

Wyborn (1) adapts Jasanoff's (20) terminology into the concepts of vision, knowledge, process, and context to analyze the effectiveness of different co-productive governance mechanisms:

- “Context (material): the broader social, ecological, and institutional context in which each initiative is situated;
- Knowledge (cognitive): knowledge related to the science, practice, and governance of connectivity conservation;
- Process (social): the formal and informal rules shaping collective action within each initiative;
- Vision (normative): the motivations guiding collective action and aspirations of what “should be done”. (p. 60).

In the next section, I will describe how these themes will be adapted in this paper for the context of biosecurity.

### Biosecurity governance

Biosecurity means literally making life safe, from the Greek *bios* for life. In relation to agriculture biosecurity generally refers to practices that prevent the spread of pathogens onto areas where farm animals are present and/or the development of disease in animals. This section will detail examples of shared responsibility in biosecurity governance and how they have been theorized previously. Shared responsibility is taken to mean any type of collaborative public-private initiatives in animal health governance, rather than the more normative, aspirational framework of co-productive governance.

Research has analyzed a shift in recent years in different settings from “command and control” government regulation of biosecurity to shared responsibility in decision making between different actors (21–24). Shared responsibility for biosecurity governance can lead to more effective surveillance and responsiveness and include a wider range of actors than command and control mechanisms (25). In some cases, such as Australia and New Zealand a sharing of responsibility for animal health between government and industry has been taking place for several decades. A not-for-profit public company that coordinates animal initiatives was established in Australia in 1996 (26) and 1998 in New Zealand (27). The establishment of Animal Health Australia was preceded by industry commitment to and financial support of a successful programme to eradicate bovine tuberculosis in Australia (28).

In Ireland, the industry-government partnership in BVD eradication is institutionally formalized in the not-for-profit company Animal Health Ireland (AHI), established in 2009 and funded by the Irish government and industry bodies. AHI leads initiatives to tackle non-regulatory diseases (29). Before the establishment of AHI, the agriculture industry had limited involvement in coordinating disease eradication and control measures (29). In Ireland, AHI eradication schemes such as the BVD scheme are governed by a technical working group and an implementation group. The technical working group provides evidence and analysis and the implementation group is made up of government and industry stakeholders who make decisions about the design of the scheme.

In Scotland, the BVD eradication scheme is governed by a BVD advisory group made up of government and industry stakeholders (30). After the establishment of the BVD eradication scheme, a not-for-profit limited company Livestock Health Scotland was established in 2015 made up of industry and government stakeholders tasked with improving animal health and welfare in Scotland. Livestock Health Scotland is co-funded primarily by the Scottish Government and Quality Meat Scotland.

As the concept of co-production in governance has been treated at times as a panacea (31), similarly shared responsibility in biosecurity is hailed as essential for effective biosecurity governance but is under theorized as a concept and in its practical application (8). The paradigm of shared responsibility for biosecurity has been subject to criticism and contestation. Shared responsibility can be understood as a neo-liberal strategy of individualizing responsibility and risk from the government to individual citizens (32) and farmers (21, 33). Responsibility for biosecurity might be moved away from the government because government cut backs mean there is a need for other actors to take responsibility (24, 34). There may be disagreement about where responsibility for biosecurity lies: research has shown farmers maintain government should have responsibility for aspects of biosecurity in protecting farmers' ability to produce food (34–36). The legacy of government responsibility for managing animal health was identified as a barrier to adopting a new model of shared industry-government responsibility in Ireland (29). The paper will use the term “lobbier and lobbied” to refer to a model of biosecurity governance where the government take primary responsibility for animal health initiatives and industry bodies seek to influence them. Here the industry are the “lobbiers” and government are the “lobbied”.

Knowledge can be contested within the domain of biosecurity. There is extensive research debunking a “deficit model” of biosecurity which presupposes farmers and other actors operate in a knowledge vacuum in relation to biosecurity (37–39). Farmers have their own understanding of how to manage the disease on their farms (40). The previous study has explored different understandings of biosecurity between vets and farmers (41), farmers and experts (42), vets and formal disease testing guidelines (43), and different forms of veterinary epidemiological expertise (44). In keeping with the concepts of adaptive and co-productive governance, researchers have suggested the need for shared responsibility in biosecurity governance to incorporate different forms of knowledge: farmers' as well as other experts' understandings of how livestock disease is best managed (22, 45).

Rawluk et al. (8) suggest how shared responsibility in passive animal health surveillance could be redesigned to incorporate principles of adaptative governance in Australia. They argue that the current mechanisms for passive surveillance are too punitive and instead, they conceptualize adaptive biosecurity governance as co-designed with relevant communities and flexible to different forms of knowledge and changing circumstances. This would allow different groups to feel committed to achieving biosecurity aims rather than feeling they need to comply with regulations imposed elsewhere.

In Scotland and Ireland, analyzes of BVD eradication schemes have focused on costs and benefits (46, 47), modeling the optimal testing methods for achieving eradication (48) and epidemiological analyzes of the schemes (3, 4, 49). Reviews have been carried out on the voluntary phase of the eradication scheme in Ireland (50) and more recent analyzes of the design, progress, and challenges toward BVD eradication (51). Research has considered farmer experiences of the voluntary stage of the Irish scheme (52), the regulatory phases of the Scottish scheme (53), and analysis of international examples of BVD eradication (54).

A previous paper using some of the research data in this paper was published analyzing the BVD eradication schemes in Scotland, England, Wales, Northern Ireland, and Ireland as an example of policy making within the institutional void, using the concept of institutional logics (55). This paper focuses only on Scotland and Ireland and includes additional interviews with key stakeholders. The focus of Shortall and Calo (55) was primarily on theoretical understandings of animal health governance. This paper explores different themes and makes recommendations for those involved in animal health governance.

In light of this existing research, I will adapt Wynborn's (1) concept of co-productive governance concepts, described in Section Co-production, for the context of biosecurity governance. By vision, I mean the vision of how biosecurity should be carried out in relation to BVD. By “context”, I mean the social and political circumstances of the agricultural sectors under analysis, which influence the feasibility of different modes of biosecurity governance. By “knowledge”, I mean the different kinds of knowledge used in decision making in relation to biosecurity at the farm and governance levels. By process, I mean the formal and informal rules that govern the BVD eradication schemes. The results will be analyzed using these concepts to explore lessons learned from BVD eradication schemes for biosecurity governance.

## Methods

The results are based on qualitative interviews with key stakeholders involved in the BVD eradication schemes in Scotland and Ireland. Qualitative interview data is not taken as representative of the attitudes or behaviors of a group of people as may be the case with quantitative data (56). Rather, qualitative interviews are an opportunity to explore an individual's perspectives in detail to engage with the reasons and mechanisms underpinning the organization of the social world (57, 58).

Purposive sampling was used to interview people with expertise and experience in the BVD eradication schemes in Scotland and Ireland (59). Interviewees were identified through publicly available information about the schemes and then through snowball sampling. Purposive sampling was used to access people who worked for different types of organizations and were likely to have different perspectives: for instance, people from government, academia, farming organizations, and veterinarians. The snowball sampling was also a way to ensure access to a range of positions, interviewees were asked to recommend other people who were influential in the development and delivery of the schemes. An information power approach was used to specify the number of interviews needed (60). The information power approach contends that the appropriate sample size depends on sample specificity, study aim, use of established theory, quality of dialogue, and analysis strategy (60). The sample size reported in this paper was considered appropriate under this framework because the study aim was narrow in terms of focusing on lessons learned from the governance of the BVD eradication schemes, there was a relatively small pool of people eligible to be interviewed and the interviews were in depth and information rich.

Ethical approval was obtained from the James Hutton Institute Research Ethics Committee. Seven people were interviewed in Ireland and 10 in Scotland. Five Irish interviews were conducted between 2018 and 2019 and 2 interviewees in 2021. Six Scottish interviews were conducted between 2018 and 2019 and 4 in 2021. Interviewees included academics, government employees, employees of organizations involved in administering the schemes, employees of farming organizations, laboratory employees, and in Scotland employees of livestock markets and a private vet. A previous study considered the perspectives of Scottish farmers on the BVD eradication scheme (53). Interviewees were sent an information sheet about the interviews and agreed to a consent form. Interviews took place over the phone, through video conferencing software, and in person and lasted around an hour, with the shortest being 35 min and the longest 115 min. Interviews were semi-structured, following an interview guide but adaptable to ask improvised follow-up questions about particular lines of discussion. The interview guide covered questions about the interviewees' background, the nature of their involvement in the BVD eradication schemes, what they thought worked well in the development of the schemes, what the challenges were, and what could be improved on in the future. Interviews were audio recorded and transcribed by a third party and are fully anonymised in the results section.

Thematic analysis was carried out (61). The thematic analysis involves “coding” the text for “patterns of shared meaning across the dataset” [(61) p. 592]. The software Nvivo 12 was used to code the data. The goal of thematic analysis is to be both systematic and rigorous in how the data is analyzed while recognizing that the process is fundamentally a creative and interpretive one (62). In “reflexive thematic analysis” (62), the researcher is an active participant in producing the themes rather than objectively “discovering” them. Analysis was primarily deductive: the co-productive governance theoretical framework was identified before analysis took place and transcripts were coded into sub-themes under the four concepts described in Section Theoretical framework. Sub-themes within these concepts were identified inductively through the coding process. The reflexive thematic analysis involves creating and editing codes in an iterative process to make sure they are broad enough to encompass different instances while also specific and meaningful (63). Codes are then analyzed for the important themes which will be used in the final analysis (63).

## Results

### Vision

In Wyborn's (1) terminology, vision means “the motivations guiding collective action and aspirations of what “should be done” (p. 60) In relation to the vision of what eradication should look like, stakeholders described the importance of creating a coherent “narrative” about the disease itself and how eradication would take place. BVD was framed as a “straightforward” disease because tests are reliable and transmission pathways are known, and the logic behind eradication in both countries was to remove PI animals. A narrative about eradication involves clear messaging about goals, progress, and endpoints which can be communicated to the actors involved.

Scotland: You need to be clear all the time, where you're going, why you're going, and when.

The first step in both countries was deciding on an approach and communicating that to vets and farmers:

Ireland: And the first output was really an educational leaflet, that we produced, it was peer reviewed externally, the people on the group were all experts, or specialists, in relation to BVD in terms of research. And that was distributed to vets and farmers, so there's an agreed approach.

This narrative needed to replace alternative existing narratives among vets and farmers about the best way to tackle BVD.

Scotland: We discovered, in the early days, through a series of roadshows which were carried out by [individuals involved in the scheme] when they were speaking to vets and farmers, there was some lack of detailed knowledge. So although the science was all there, even the veterinarians didn't always have a really firm and robust grasp of the disease, where its weak points are and how to manage it. There was still a notion amongst some of the vets and farmers that it's a bit like when we were kids, and you had chicken pox parties: it was better for everybody to get chicken pox at the same time. And you get immunity after that and you move on. You know, I think these are maybe some of the reasons why it wasn't dealt with by the industry themselves. The industry being vets and farmers and their vets.

In relation to the narrative, a Scottish stakeholder acknowledges that more formal targets and reviews to assess the progress of the eradication scheme would be helpful.

Scotland: I think that it's more a planning issue which I think, in retrospect, you know, having clearer targets and clearer milestones which you communicate with everybody but also review points, accept that every 3 years or something, 4 years, you actually take stock of where you are and actually, be willing, at that point, to change. […] it maybe makes sense then, to involve the wider industry in that review so they can actually confirm the positives that you've achieved but also, if there are negatives, actually, you know, formalize them or point them out.

Part of establishing a scheme “narrative” is deciding from the outset what the endpoint of eradication looks like. What is the definition of freedom from BVD and how will that be achieved?

Scotland: And the other things I would say about the scheme is that there has been, and I'm as guilty of this as anybody from the start, there was not a good picture made of what eradication looks like. So what are we expecting to achieve and what does it mean for everybody? So, eradication was just, we get rid of the virus, no more BVD. But what else do you need to do? What, what controls and surveillance is required? There was no real discussion on that, although we've tried to have those discussions more recently but that was one thing that was missing in the early days was this, “Well, what does this actually look like?”

Thus, the vision of co-productive governance of BVD eradication was about creating a clear and agreed upon a narrative about the characteristics of the disease, the mechanisms, and the goals for eradication. This narrative might involve replacing stakeholders' existing understanding of how BVD operated. Some of the lessons learned were that the creation and communication of the narrative could be reviewed more regularly with the industry and could include from the outset what eradication would look like.

### Context

Wyborn (1) describes context as “the broader social, ecological, and institutional context in which each initiative is situated.” The stakeholders described how the legacy of existing relationships in the country's agricultural sector shapes the design of the scheme. Interviewees listed multiple epidemiological, economic, and logistical reasons why the testing regimes in both schemes were chosen, which have been reiterated in the literature (50). Among the reasons were the social and relational implications of different testing methods. A key stakeholder detailed how in Ireland the tissue tag testing method was chosen over a serological blood test because farmers could implement it themselves without the involvement of the vet.

Ireland: But again, there was a strong, I suppose the political piece was as much, […] putting it in the control of the farmer as requiring the vet to be on farm, so, there was all of those pieces, I suppose, that fed into why a tag programme rather than a serology programme.

Ireland: A serological based programme would have probably have to have been veterinary practitioner led. And there was quite a bit of antagonism among the farming community to a veterinary led programme and they basically wanted to take, in so far as possible, to take vets out of that equation and tagging did that.

Stakeholders explained that there was a legacy of tension between farmers and vets in relation to the control of bovine Tuberculosis (bTB), with farmers being unhappy with what they saw as benefits accruing to vets through the scheme:

Ireland: There's been a little bit of tension between the farmer body, and the vets, historically and it has to do with tuberculosis and that's an interesting story in itself.

This stakeholder states that this tension has been addressed and vets have become more involved in later phases of the Irish eradication scheme in herd investigations for persistently infected animals. Another stakeholder reflects that it is important to keep all stakeholders involved and interested in the scheme, even if their active involvement is not needed in all stages.

Ireland: I suppose it applies to any stakeholders that, that you should have a reasonably long term view of the design of the programme and then say well, “I don't need them now, but at this stage, I will need them, so I want to keep them happy or keep them feel valued at this stage.”

In contrast, Scotland had become bTB-free before the start of the BVD eradication scheme and the option of vets carrying out blood tests on the farm was seen as a way to facilitate vets visiting hard-to-reach farms and encouraging vet/farmer interactions.

Scotland: Not long before BVD started, we had achieved official TB free status. That meant that there was some additional resource available, it also meant that there were potentially fewer vets visiting beef farms in Scotland. And one of the things we knew was that there's a problem with vets in remote areas visiting livestock holdings. So, as a small step to redress that, requiring a blood testing meant that you had to have a vet go on to a farm, so vets would then be paid, they would be part of the scheme, and it was intended to stimulate or continue farmer-vet interactions.

We can see that the context of existing relationships within the agricultural sectors influenced the design of the scheme. The legacy of the bTB eradication scheme in Ireland was perceived to have created tensions between vets and farmers so the BVD eradication scheme was designed to involve tests carried out by the farmer. In contrast in Scotland, there is no active bTB eradication scheme so the BVD eradication scheme was seen as an opportunity to stimulate more vet-farmer interactions through the serological test carried out by vets. Thus, the schemes were not only designed within the context of existing relationships but sought to influence those relationships as well. We will return to this point in the discussion.

### Knowledge

Wyborn (1) described knowledge as “knowledge related to the science, practice, and governance” (p. 60). Both eradication schemes in Scotland and Ireland (29) aim to make decisions based on science. BVD was identified as a priority disease in an expert Delphi study carried out by AHI which included farmer input (64) and an economic analysis established the rationale for eradicating BVD in Ireland (46) and Scotland (47).

As well as being the basis for decision making, science also gives legitimacy to actions taken in the design of BVD eradication schemes.

Ireland: I realized, what we needed was a model. A mathematical model, so that when the minister would ask that question, we would be able to say, “Well, here's the model, and we expected to at this stage in the eradication programme based on the modeling, but we're not, and the reason is this.” On reflection, we would have, ideally, had a mathematical model for BVD, right at the onset.

Scotland: Researchers came up with figures about what the disease was actually costing the national herd. This, at least, supported the idea that the disease should perhaps be addressed on a national basis.

In Ireland, the scheme was originally planned to involve 3 years of tissue tag testing followed by a move to a cheaper serological testing regime but the duration of the tissue tag testing had to be extended.

Ireland: The initial problem was, there were only going to be 3 years of test, of ear notch testing, we found that, due to various different reasons, principally PI retention, that that had to be extended.

There was disagreement among stakeholders in Ireland about why reality did not live up to the expectations of a 3-year tissue tag programme. This interviewee mentions “PI retention”: that is, farmers holding on to PI animals against epidemiological advice to cull them. Another interviewee describes this problem in relation to the original plan of 3 years of tissue tag testing.

Ireland: But that's been a big debate, the farmers are really saying, “You told us we would have been clear after 3 years, and look, it's all your fault.” But they understand that, and I think, there's been a lot of progress made. But then, on reflection… we probably underestimated the suckler farmer piece.

By the “suckler farmer piece” this stakeholder means that many farmers, particularly suckler cow farmers, held on to PI animals rather than culling them in accordance with the guidance of the scheme (3, 4, 50, 51).

Another stakeholder points out that when they produced a model, it *did* validate the 3-year target, but it relied on farmers removing PI animals promptly.

Ireland: So we had said, you know, 3 years would get us to that point. And we didn't get there in 3 years, but that was because not everybody did the right thing. But the model very clearly demonstrated that, if that had have been done, that we would have got to, where, you know, logically, so you could say you were getting to, because any herd that's got PIs, you would, 2 years were sufficient to clean it up, if you go at it.

If farmers had removed PI animals, then the model would have been accurate. Other stakeholders felt that the 3-year target was a misjudgement on the part of AHI, which damaged the credibility of the eradication “narrative”:

Ireland: That was probably the biggest mistake, because AHI lost serious credibility in the minds of farmers because, in farmers' minds, they were sold a 3 years programme by AHI, which commenced in 2013, and is now going to run to at least 2020.

A later modeling exercise showed only 25% of herds stood to benefit financially from a move from tissue tag testing to serological testing, and those farms were predominantly dairy herds (48). This meant that the target of 3 years of tag testing was not considered optimal.

This disagreement can be seen as an example of a debate becoming “scientized” (15), as described in the introduction: discussions about governance and decision making turn into arguments about science. There are two different stories about why the 3-year target was not reached: because of the “incorrect” premises the target was based on, or because farmers did not follow epidemiological advice to remove PI animals. In one story, decision makers are at fault for not anticipating the actions of farmers. In the other story, farmers are at fault for not listening to the advice of decision makers. Should the expectations of those with epidemiological expertise take into account farmers' lived experience, or should farmers better adapt to those expectations and voluntarily get rid of PI animals?

The story was also more complicated because stakeholders with epidemiological expertise did not uniformly back the 3-year target. An interviewee expressed the view that though the initial 3-year target was not based on any scientific study, scientists ended up being held responsible for its failure.

Ireland: There was considerable unease among several TWG members about the 3 years of tagging followed by 3 years of serological surveillance, particularly the suggestion that the TWG endorsed that approach, when in fact several members felt it was non achievable and was overly optimistic. Unfortunately, that pessimism was well founded, as events later transpired.

When the 3-year target was not met, “science” was blamed, even though there was not necessarily a scientific consensus behind the target. This shows the complicated role of scientific input in co-productive governance. As described in Section Biosecurity governance, science can be used to inform decisions, give legitimacy to positions (16), and be held responsible if decisions go wrong.

Science was not considered neutral and a-political in the Irish BVD scheme. An interviewee described tensions in the production and use of science in decision making.

Ireland: I think this is at a lower level, so it's within highly politicized discussions and often what the scientists have to say is not helpful or certainly doesn't support people's views so, I think it's a little bit closer, and I think being an honest broker is more, is harder to achieve.

Ireland: And in a model of the lobbied and the lobbier, I guess, [scientists] were probably seen to be linked to the government more.

The BVD schemes were industry led and involved the industry making decisions about a non-regulatory disease (51). To consider this as a form of co-productive governance means that it is different from a previous model of “the lobbied and the lobbier”: where industry lobby government to take action and bear the cost for pursuing improvements in animal health. According to the interviewee above, within this older “lobbied and lobbier” model of governance, scientists were seen as linked closely to the government. Interviewees stated that historically agricultural organizations have been effective and important lobbyists in Ireland. The legacy of this model continues into a co-productive governance model despite aspirations that the science would be understood as an objective and neutral resource for decision making.

But according to interviewees the production and use of science did change during the scheme. In relation to the question of whether farmers “should” have culled PI animals in line with the optimal situation modeled, interviewees reconciled this ideal situation with the farmers' own perspective:

Ireland: I think a lot of that had come down to the fact that, for these beef farmers, all their income was contained in these calves. You know. For a dairy farmer, it mightn't have been so palatable to destroy a calf, but, at least, it isn't his sole income, his main income was left intact through the sale of milk, whereas with a beef farmer, all the income for that cow was gone.

Beef farmers in particular found it difficult to cull PI calves because their calves are their future income. Other interviewees also recounted the situation from the farmers' point of view.

Equally a stakeholder states that research showing the necessity of disposing of PI animals for the success of the scheme (3, 48) was useful in convincing farmers that removing PIs was important and justified further measures to encourage this in the scheme.

Ireland: It's been slower than originally planned, and that's because of issues like PI retention, and farmers understand, because of the modeling, farmers understand that those issues probably set us back 3 or 4 years. And, they're aware of that and that was the reason they made hard decisions to start to put pressure on farmers who are not co-operating.

Industry groups became more involved in the formulation of the science as the scheme progressed.

Ireland: So, the questions they ask now are, are very hard to answer, and they're much harder than previously, I guess, science could be fairly siloed, so you could, you know, do a piece of study on PI retention or whatever, and that's fine and that's important and it provides really valuable information. But it doesn't always answer the questions the farmer organizations wish, and I think the questions they ask are hugely well-informed, you know, these are very intelligent people, but they ask hard questions and the questions really relate to cost and time, and success, I guess.

Here, industry input is framed as enhancing the science. This moves beyond the model of “lobbier and lobbied” where the role of the industry bodies may have been to challenge the science that they saw as reflecting government interests. An interviewee states that decision making about the development and design of the Irish scheme was difficult, but ultimately this was potentially beneficial for pursuing eradication and avoiding “group think”.

Ireland: Because there's nothing gets decided here without it being examined, from every aspect and wrangled over and pushed and pulled and, that brings an element of stress testing to the process which is certainly stressful and testing. [laughter] But actually, it does make the decisions more robust. And I think that that is a good thing. Sometimes, there could be too much, there's a danger, perhaps, of, well, again, we'll call it group think at least, “we'll all go with the flow”.

While the Scottish scheme was also informed by science: scientists also carried out work modeling the financial costs of PI retention in Scotland (65), interviewees did not describe the politicization of the science in Scotland or advisory group input into the design of the science.

Interviewer: And how involved are the steering group in the science? Do they ask for the scientific outputs about it, do they ask you for particular studies to be done?

Scotland: They, they haven't specifically requested, I don't think. They quite like hearing about it. […] I think the, they'd probably trust us to get the, to kind of get the information and interpret it and say to them.

According to interviewees, in Scotland, science is not fought over and contested compared to Ireland. Thus, one lesson learned from the BVD eradication schemes was that the legacy of past models of biosecurity governance might mean that science may not be considered neutral and value free within a model of co-productive governance. We will return to the differences between the countries in relation to the intersection of science and politics in the discussion.

### Process

Wyborn (1) defines process as “the formal and informal rules shaping collective action within each initiative.” Both schemes in Ireland and Scotland were “industry led” which fits with the co-productive governance vision that different actors are involved in co-producing outcomes. In both countries, BVD eradication had not been achieved at the time the research was carried out but the models of co-productive governance that the schemes were based on were seen as an overall success by interviewees:

Ireland: I think the model that we have is working well. […] And the government recognizes this, and recognizes also they do not have the resources to tackle that themselves, and also that, the industry needs to take ownership of some of these issues and do something about it. And I think that we found that, with Animal Health Ireland, it's working well in that relationship […] So, yeah, I think it will continue in this system, this private public partnership in the future with other programmes for sure.

Many interviewees in Ireland and Scotland expressed similar views about the success of the schemes. Interviews also showed how the livestock “industry” was not one homogenous entity with homogenous goals. This could complicate the achievement of scheme outcomes because all stakeholders might not be equally invested in achieving scheme outcomes. An interviewee points out how some livestock farmers (those who bred animals) bore the majority of the costs of the scheme, while others (those who fattened cattle for slaughter) also benefited.

Ireland: Because, if you look at the dynamic, all of the costs associated with the BVD eradication programme are, are targeted at the breeding farms, the suckler and the dairy farms. They would always have been able to sell on these animals as calves, that they would be moving them as young animals, so they were rarely identified as sick animals, or rarely caused huge problems on these farms. And effectively, it was the rearing or the feeding or the fattening farms, that were having the problems of BVD and the production losses. So, them guys got the benefit but the other guys actually incurred the cost.

“Famers” includes a diverse group of people, some of whom may resist the aims of the scheme for different reasons. A stakeholder in Ireland reflects that though the scheme is industry led, i.e., it involves representatives of different farming organizations, this does not mean all farmers agree with the aims of the scheme.

Ireland: but you have to just be aware of that, don't expect too much when you're designing [laughs] these programmes that you, even there might be stakeholder led or industry led, don't expect your man in the field to act on that, on those principles.

Similarly in Scotland:

Scotland: There was some farmer opposition to the scheme which you've probably discovered, one or two letters to the farming press.

A particular challenge was involving “hard to reach” farmers who resisted attempts to communicate and enroll them in the scheme.

Scotland: I think reaching the hard-to-reach farmers is the, that's the massive challenge. That's something kind of […] by definition, they don't read the farming press, they are not interested in official correspondence, they're unlikely to phone a helpline and they don't have much to do with their vet, so it's hard, really hard to know other than going round and tapping the door, and how do you, how do you get them on board?

An interviewee describes different types of farmers who might resist what they perceive as outside interference on their farm.

Scotland: At one side of them you've got the “I know better than any of these bloody scientists, I've been doing this for 50 years, go away and leave me alone, stop interfering!” At the other end there are the people who are really, really good farmers who have worked out their own way of having a top-notch herd and it doesn't necessarily coincide with what the powers-that-be are telling them and they object because they, probably justifiably, feel that they run a very tight ship and are capable of dealing of these things, their way, themselves.

The interviewee's wording of “powers-that-be” suggests that industry led schemes may not break down perceptions of power differentials in all cases and will not necessarily result in all farmers taking ownership of the scheme. This will be explored in more detail below.

## Discussion

This study sought to analyze key stakeholders' views on the design of BVD eradication schemes in Scotland and Ireland using a theoretical framework of co-productive governance. The results drew on Wyborn's (1) co-productive governance theoretical framework of visions, context, knowledge, and process. The section on “vision” described the importance of creating a clear “narrative” about the disease itself and the goals of the eradication scheme. The importance of simple and consistent messaging for stakeholders around the eradication of BVD has been highlighted in relation to the Swedish example (54) and in Switzerland (66). Interviewees described how embedding the narrative might involve replacing existing understandings of BVD in some cases. Several interviewees recognized that the “narrative” might have to replace farmers' own understanding of the disease. Thus, they did not subscribe to the' deficit model' that promoting animal health is just about experts giving farmers more information (37–39). One interviewee described a lay epidemiological “chickenpox” model of the epidemiology of BVD that exists among farmers and vets at the beginning of the scheme in that they believed exposure to BVD could mean an animal then had immunity to future exposure, and so exposing animals to infection was the best course of action. Other research has also described practices of managing immunity on pig farms which differ from prescribed advice (67). This “chickenpox” model may have allowed farmers and vets to “live with” BVD (68). That is, to keep farming when the disease is one among many challenges farmers face (69). But it was not a helpful model to motivate farmers and vets to eradicate BVD. Thus, the BVD eradication schemes involved creating new narratives about BVD as a disease that could and should be eradicated. Previous research with farmers in Scotland has shown some endorsement of the goals of the BVD eradication scheme (53), and that farmers in Ireland were satisfied with the implementation and communication of the voluntary phase of the scheme (52).

One of the lessons learned was about further improving the “narrative” about the eradication scheme. Interviewees stated that the end goal of what eradication would look like could be further specified in advance, and the industry could be further involved in reviewing the scheme goals and milestones. This fits with Wyborn's (1) aspiration for co-productive governance to involve “critical, inclusive, and reflexive practice” (p. 59).

The results showed how the context of existing relationships within the agricultural sector influenced the design of the scheme. In Ireland, there was a desire by some for testing to be carried out by farmers so it would not replicate the bTB eradication scheme which cost farmers vet fees. Whereas in Scotland, in contrast, bTB is not an endemic disease so vet-led testing was seen as a potential opportunity for vets to visit farms.

Existing relationships were among the many other epidemiological, logistical, and economic reasons for different testing methods being chosen. A commentary on the BVD eradication schemes in the British Isles recognized that eradication schemes are designed based on the circumstances of a specific country (70). Co-productive governance involves navigating the competing interests and values of different individuals and organizations (12). Experiences from shared responsibility governance in Australia have shown that different organizations bring their own priorities and history to bear on biosecurity governance (34). But organizations are able to deploy their own perspectives in a flexible way to work together (71). Similarly, in Ireland interviewees described the allowances made to enable farmers to be in charge of the testing method, while supporting the greater involvement of vets as the scheme progressed.

An additional dynamic identified in the results was how the schemes could be designed to influence relationships and achieve multiple biosecurity objectives in promoting vet visits on the farm in the Scottish scheme. The vet-farmer relationship is seen as key to improving on farm biosecurity (23). But government funding of veterinary services has been reduced in the 20th century thanks to neoliberal policies (23). It is interesting to note that this was an industry led biosecurity scheme taking on the role of promoting vet-farmer interaction which was previously a role of government in the 20th century (72).

In terms of lessons learned the results show that it is difficult to engage all actors in biosecurity governance when initiatives are developed with the legacy of existing relationships and tensions. Different actors might play more or less important roles at different stages of eradication schemes. Future research could explore if the serological testing option in Scotland did in fact lead to more vet-farmer interactions that subsequently improved herd health.

Literature on shared responsibility in biosecurity has not considered in detail the production and use of science within this governance regime. As described in Section Co-production science can have different functions in relation to government: either instrumentally to inform policy making, or to legitimize or substantiate policy positions (16). Interviewees described science being used in both of these ways in the BVD eradication schemes. A Delphi study of disease priorities (64), economic analyzes in Scotland (47) and Ireland (46), and the modeling exercise in Ireland (48), among other pieces of research, were used instrumentally to inform the decision to establish BVD eradication schemes and the design of the schemes.

Interviewees also described the legitimizing function of science: the initial economic analyzes legitimized the goal of eradication for the industry as a whole, and in Ireland, later scientific work legitimized measures to tackle the retention of PIs. Boswell (16) states that knowledge can have a legitimizing and substantiating function when there is contestation over policy and more research is needed to bolster credibility. This fits with the description of the intersection of science and decision making in the Irish context, where there was contestation over the design of the scheme. Interviewees described disagreements about a failure to meet an initial target of 3 years of tissue tag testing before moving to a lighter touch testing regime. There were different explanations for why this happened: that the target was based on false premises about the costs of different testing regimes for different groups, and/or scientific consensus on the 3-year target was not reached, and/or that farmers did not cull PI animals in line with expert advice which slowed down the progress of the scheme.

As the interviewees described, the role of science in the Irish scheme was more politicized and subject to debate than the account given by Scottish interviewees. According to one interviewee, the contestation over science in Ireland was linked to the legacy of a “lobbier and lobbied” model, where science was produced by the government and the role of lobbying bodies was to challenge the science if it was not in their interest. Interviewees put this down to the historic importance of agricultural lobbying organizations in Ireland. Sarewitz (15) refers to this as debates becoming “scientized”: debates about values and politics can get displaced to the realm of science, with one side arguing for one body of scientific research and another highlighting the uncertainty in the science or championing an opposing body of research. Additionally, indeed the legacy of government responsibility for biosecurity has been pointed to as a hurdle to moving to a new model of biosecurity governance in Ireland (29).

Within a context of groups working together in a co-productive governance model, it was a challenge for science to be seen as “neutral” and produced in the interests of all organizations rather than just the government. This idea that science and government are linked has a long history. The development of modern European states in the 18th century was predicated on the government's ability to gather information about their population in order to standardize and regulate their activities (73, 74). The 20th century saw an expansion in the state funding of science to foster productivity and the competitive development of society (75). Thus, conceptually, there are reasons why science is linked to the government.

Previous analyzes of the use of scientific research in biosecurity governance have shown how science is used not only to legitimize certain positions but a step further, to “depoliticize” them (76, 77). That is, when problems are framed in overly technocratic terms, it only becomes possible to contribute to debates through technical, scientific language, and wider political and value-based questions are not openly discussed (76, 78).

In the Irish scheme, however, the science had instrumental and legitimizing functions but was not used further used to depoliticize debates. That is, science was not used as a tool to exclude certain groups or perspectives. Rather, there was a rapprochement between those who considered their perspective to be aligned with the science and those who challenged it. Interviewees recounted how they understood why beef farmers might hold onto PI animals, going against the epidemiological advice given in the scheme. Additionally, industry stakeholders became more involved in the setting of scientific questions. Interviewees state ultimately the decision-making process was difficult, but the contestation made decisions more robust. This is in keeping with a co-productive governance perspective that the involvement of multiple actors in knowledge production enhances decision making (19).

Thus, the lessons learned from interviewees' accounts were about being aware of the legacy of the role of science in previous forms of biosecurity governance, including all actors in devising scientific questions and taking a flexible approach that involves trying to see challenging or controversial aspects of the scheme from different perspectives.

Finally, the results showed how “industry” or “farmers” were not a homogenous category and while industry involvement in biosecurity might have many benefits it is not a panacea for achieving consensus or enrolling all farmers in a goal. Interviewees described resistance from certain farmers and reluctance to be involved in the scheme despite it being industry led. This accords with previous findings that industry led schemes might not find favor with all farmers. For example, risk based trading to control the spread of bovine TB in New Zealand was more or less applicable and appropriate in different contexts with their own unique disease concerns, geography, and market circumstances, even though the rules were co-produced with industry (27). Compliance with certification schemes or corporate biosecurity rules can engender resistance in farmers who object to the amount of paperwork and audits they are subject to, so compliance becomes about meeting administrative goals rather than complying with biosecurity goals themselves (79, 80). In the case of BVD, these problems did not disappear when the scheme was designed and administered by a public-private group rather than by the government (80) or private industry actors (79). Thus the lessons learned suggest that there is a need to be aware of who is, or is not, benefitting from biosecurity iniatitives, and recognize that co-productive governance is not a panacea for uniting disparate actors behind biosecurity goals.

The study focuses on two countries to highlight similarities and differences in how co-productive governance operates in different contexts. The results could be applicable to different countries and biosecurity governance regimes. The “vision” findings about the importance of creating a narrative about eradication and focusing on the end goals which are continually revisited are widely applicable. The result that no governance regime is a panacea for enrolling all actors is also useful to consider in a different setting. Findings about relationships between actors within biosecurity governance and the use of science will be highly dependent on the specific circumstances. Further research could focus on how best science can be designed and used to best inform disease eradication schemes.

Interviews were carried out with people who were involved in the development and/or implementation of the eradication schemes and so had a stake in the success of the schemes. This meant that more critical perspectives may be excluded, for instance, people who maintain this type of disease eradication approach are not optimal. In addition, an interviewee in Scotland pointed out that involvement in the governance of the BVD eradication scheme is not paid and so may exclude people, such as farmers or vets, who would need financial remuneration to support their contribution. Thus, the interviews represent the views of a specific group of people directly involved in the eradication schemes. See Shortall and Brown (53) for research on the experiences of farmers in Scotland with the eradication scheme.

## Conclusion

Findings about lessons learned from the design of BVD eradication schemes in Scotland and Ireland were analyzed using a co-productive governance theoretical framework of vision, context, process, and knowledge. In relation to vision, the results show the importance of creating a clear narrative about the goal of disease eradication schemes, which may incorporate or replace existing vet or farmer “narratives” about a disease. Regarding context, it is difficult to engage all actors in biosecurity governance, when initiatives are developed with the legacy of existing relationships and tensions. In relation to knowledge, the results showed the importance but political complexity of basing decisions on scientific research. Involving industry stakeholders in setting scientific questions to inform the design of the scheme could be an effective way to enact co-productive governance. Additionally, with reference to the process, while interviewees were enthusiastic about future prospects for industry and government working together to achieve biosecurity goals co-productive governance is not a panacea for enrolling all actors in biosecurity goals. The findings are applicable to other contexts where public-private governance of biosecurity is underway or aspired to.

## Data availability statement

The datasets presented in this article are not readily available because the interviews were sensitive in nature and the interviewees were assured that data would not be shared outside of the project team. Queries about the dataset should be directed to OS, orla.shortall@hutton.ac.uk.

## Ethics statement

The study was reviewed and approved by James Hutton Institute Research Ethics Committee. The participants provided their written informed consent to participate in this study.

## Author contributions

OS conceptualized the research question, carried out the interviews, analyzed the data, and wrote the paper.

## Funding

The work was funded by the Scottish Government Rural and Environment Science and Analytical Services Division, as part of the Center of Expertise on Animal Disease Outbreaks (EPIC; Edinburgh, Scotland).

## Conflict of interest

The author declares that the research was conducted in the absence of any commercial or financial relationships that could be construed as a potential conflict of interest.

## Publisher's note

All claims expressed in this article are solely those of the authors and do not necessarily represent those of their affiliated organizations, or those of the publisher, the editors and the reviewers. Any product that may be evaluated in this article, or claim that may be made by its manufacturer, is not guaranteed or endorsed by the publisher.
